# Performance of a knowledge‐based planning model for optimizing intensity‐modulated radiotherapy plans for partial breast irradiation

**DOI:** 10.1002/acm2.13506

**Published:** 2021-12-22

**Authors:** Amy Frederick, Michael Roumeliotis, Petra Grendarova, Sarah Quirk

**Affiliations:** ^1^ Department of Physics and Astronomy University of Calgary Calgary Alberta Canada; ^2^ Division of Medical Physics Tom Baker Cancer Centre Calgary Alberta Canada; ^3^ Department of Oncology University of Calgary Calgary Alberta Canada; ^4^ Division of Radiation Oncology Grande Prairie Cancer Centre Grande Prairie Alberta Canada

## Abstract

**Purpose:**

To evaluate a knowledge‐based (KB) planning model for RapidPlan, generated using a five‐field intensity‐modulated radiotherapy (IMRT) class solution beam strategy and rigorous dosimetric constraints for accelerated partial breast irradiation (APBI).

**Materials and methods:**

The RapidPlan model was configured using 64 APBI treatment plans and validated for 120 APBI patients who were not included in the training dataset. KB plan dosimetry was compared to clinical plan dosimetry, the clinical planning constraints, and the constraints used in phase III APBI trials. Dosimetric differences between clinical and KB plans were evaluated using paired two‐tailed Wilcoxon signed‐rank tests.

**Results:**

KB planning was able to produce IMRT‐based APBI plans in a single optimization without manual intervention that are comparable or better than the conventionally optimized, clinical plans. Comparing KB plans to clinical plans, differences in PTV, heart, contralateral breast, and ipsilateral lung dose–volume metrics were not clinically significant. The ipsilateral breast volume receiving at least 50% of the prescription dose was statistically and clinically significantly lower in the KB plans.

**Conclusion:**

KB planning for IMRT‐based APBI provides equivalent or better dosimetry compared to conventional inverse planning. This model may be reliably applied in clinical practice and could be used to transfer planning expertise to ensure consistency in APBI plan quality.

## 1 INTRODUCTION

Hypofractionated whole‐breast irradiation (WBI) and accelerated partial breast irradiation (APBI) have been investigated for early‐stage breast cancer in multiple phase III clinical trials over the last two decades.^1^, ^2^, ^3^, ^4^, ^5^, ^6^, ^7^ Moderate hypofractionation for WBI, delivering 40–42.5 Gy in 15–16 fractions over 3 weeks, has become an international standard.^8^ Recent studies^3^, ^4^ have shown that five‐fraction regimens for WBI are also safe and effective, and are applicable to APBI. Five‐fraction regimens for WBI and APBI have been rapidly adopted during the COVID‐19 pandemic and are likely to continue being offered moving forward.^9^, ^10^

APBI is progressively being used to treat early‐stage breast cancer as an alternative to WBI, as it offers much lower normal tissue doses.^11^ Long‐term outcomes show consistent and low ipsilateral breast tumor recurrence rates for APBI, but with mixed toxicity and cosmesis profiles.^5^, ^6^, ^7^ It is hypothesized that changes in planning techniques and dose constraints may alter the therapeutic ratio of APBI regimens and contribute to the conflicting complication profiles.^5^, ^6^, ^7^, ^12^, ^13^, ^14^, ^15^, ^16^

The most common techniques used to deliver external beam APBI are three‐dimensional conformal radiotherapy (3D‐CRT) and intensity‐modulated radiotherapy (IMRT). Several APBI planning studies^17^, ^18^ have shown that IMRT improves dose conformity, dose homogeneity, and normal tissue sparing compared to 3D‐CRT. These improvements may come at the cost of an increase in the number of monitor units and low‐level radiation exposure of normal tissues,^17^, ^18^ but secondary cancer risk remains lower than for WBI.^19^, ^20^ Furthermore, there is strong clinical evidence supporting IMRT as a technique to deliver APBI.^7^ However, inverse planning can be time‐consuming, resource intensive, and subjective.^21^ Dose–volume constraints are guided by clinical experience and recommended values from the literature.^22^ Geometric variations in patient anatomy lead to large patient‐to‐patient variation in organ at risk (OAR) sparing and the need for additional dose control structures. As a result, the final plan quality is variable and dependent on time constraints, available resources, and the planner's (or institution's) experience.

Knowledge‐based (KB) planning is a data‐driven approach to inverse optimization that has been shown to improve planning efficiency, consistency, and quality compared to conventional inverse planning.^23^, ^24^, ^25^, ^26^ KB planning aims to generate the best plan based on historical, high‐quality treatment plans. RapidPlan (Varian Medical Systems, Palo Alto, CA) is a commercial KB optimization engine that uses geometric and dosimetric features from a library of treatment plans to estimate the achievable range of OAR dose–volume histograms (DVHs) for new patients.^27^, ^28^ From this prediction, dose–volume constraints and priorities are generated to drive optimization of the new plan. The goal is to produce a plan of similar quality to those used to train the RapidPlan model, with minimal human interaction.

RapidPlan has demonstrated the ability to create improved or equivalent plans for WBI^24^, ^26^ but there are no published experiences for APBI. Our institution's experience in developing a five‐field IMRT class solution beam strategy^29^ and more rigorous dosimetric constraints^30^ compared to major phase III trials^5^, ^6^, ^7^ was used to train and validate a RapidPlan model for left‐ and right‐sided APBI. This study reports on the dosimetry achievable with large‐scale retrospective application of this RapidPlan model, and compares the results to conventional clinical plans and dosimetric constraints from phase III APBI trials. The five‐field IMRT class solution beam strategy^29^ and this RapidPlan model are useful tools for institutions looking to offer APBI as a routine treatment option for appropriately selected early‐stage breast cancer patients.

## 2 METHODS

### 2.1 Patient selection and clinical planning

A total of 184 early‐stage breast cancer patients who received APBI (27 Gy in 5 fractions) as part of phase II prospective clinical trial^31^ (the ACCEL trial) were selected for model training and validation. This study was determined to be of minimal risk and consistent with a quality improvement project using A pRoject Ethics Community Consensus Initiative (ARECCI) screening tool^32^ provided by the Health Research Ethics Board of Alberta. A formal research ethics board review and approval were not required according to institutional mandate.

Planning and treatment protocol details are published elsewhere.^29^, ^31^ All patients underwent a free‐breathing CT simulation (Philips Big Bore, Philips, Andover, MA) in the supine position on a standard wing board with both arms raised. CTs were acquired with 120 kVp, 200 mAs, and a slice thickness of 3 mm. The seroma was delineated by the treating radiation oncologist, based on the seroma/surgical cavity and surgical clips visible on the CT. The clinical target volume (CTV) is the seroma plus a margin of 10 mm, cropped to the chest wall and 5 mm inside the body contour. The CTV is expanded by 7 mm in all directions to create the planning target volume (PTV). A dose evaluation volume (DEV), used to evaluate target coverage, is defined as the PTV trimmed to the chest wall and 5 mm inside the body contour. Contoured OARs include ipsilateral and contralateral breast^29^, ^31^, ipsilateral lung, and the heart.^33^

A clinical IMRT plan was created for each patient by a certified dosimetrist using the five‐field planning strategy outlined by Quirk et al.^29^, which achieves highly conformal dose distributions with improved OAR sparing. Key components of this planning strategy include: gantry and couch angle class solutions for left‐ and right‐sided breast seroma locations, clearance charts of permissible gantry and couch angle combinations based on patient body habitus and seroma location, and rigorous dosimetry guidelines^29^, ^30^ (Table 1).

**TABLE 1:** Planning constraints (minor variations) used in this study compared to those from phase III trials

Planning constraints for this study^30^	NSABP B39/RTOG 0413^5^	RAPID^12^	Florence^7^
DEV and prescription			
D98% ≥ 95%	V90% ≥ 90%	V95% = 100%	V95% = 100%
27 Gy/5	38.5 Gy/10 BID	38.5 Gy/10 BID	30 Gy/5
PTV			
CI < 1.2 (1.2–1.4)			
D_1cc_ < 107%	*D*_max_ < 120%	*D*_2cc_ < 107%	*D*_max_ < 105%
Ipsilateral breast			
V50% < 40% (40%–60%)	V50% < 60% (60%–65%)	V50% < 50% (50%–65%)	V50% < 50%
V95% < 15% (15%–25%)	V100% < 35% (35%–40%)	V95% < 25% (25%–35%)	
Contralateral breast			
V3% < 3%	*D*_max_ < 3%	*D*_max_ < 3%	*D*_max_ < 3%
Ipsilateral lung			
V10% < 20% (20%–25%)		V10% < 20% (20%–25%)	
V30% < 10% (10%–13%)	V30% < 15% (15%–20%)	V30% < 10% (10%–13%)	V30% < 20%
Heart (right‐sided)			
V5% < 5% (5%–8%)	V5% < 5% (5%–10%)	V5% < 5%	V10% < 10%
Heart (left‐sided, lower inner quadrant)			
V15% < 5% (5%–8%)	V5% < 40% (40%–45%)	V15% < 5%	V10% < 10%
Heart (left‐sided, other quadrants)			
V10% < 5% (5%–8%)	V5% < 40% (40%–45%)	V10% < 5%	V10% < 10%

Abbreviations: BID, twice daily; CI, conformity index, volume of tissue receiving at least 95% of the prescription dose divided by the volume of the PTV; DEV, dose evaluation volume; NSABP, National Surgical Adjuvant Breast and Bowel Project, RTOG, Radiation Therapy Oncology Group; PTV, planning target volume; RAPID, Randomized Trial of Accelerated Partial Breast Irradiation.

All treatment plans used five 6 MV non‐coplanar sliding window IMRT fields: four off‐axis tangents (two medial and two lateral) and a field from an anterior oblique direction. Gantry and couch angles were selected according to Quirk et al.’s^29^ class solution approach, which were adapted for patient body habitus and to maximize the angle between couch positions. Clearance charts for a TrueBeam linear accelerator (Varian Medical Systems, Palo Alto, CA) with Exact IGRT couch, detailing all possible gantry/couch combinations and account for seroma location and body habitus, were consulted to ensure a deliverable configuration. All patients were planned such that at least 98% of the DEV received at least 95% of the prescription dose. All contoured volumes and clinical plans were peer‐reviewed per trial protocol.^31^

### 2.2 RapidPlan model configuration and evaluation

The RapidPlan model configuration and evaluation processes are described in detail in the literature.^34^, ^35^ The RapidPlan model was configured in the Eclipse treatment planning system (version 15.6.06) using 64 IMRT‐based APBI plans. These plans were selected and verified to be high quality based on peer‐review of contours and beam geometry, and meeting the planning constraints in Table 1. These patients were also selected to represent a range of breast and target sizes, target positions within the breast, and body habitus (reported in Section 3; Table 4).

RapidPlan model configuration consists of data extraction and model training. Geometric and dosimetric data were extracted from the training dataset, where OAR contours are divided into four subregions: target‐overlap, in‐field, leaf‐transmission, and out‐of‐field.^34^, ^35^ For each OAR, a model is trained using a combination of principal component analysis and step‐wise multiple regression for the in‐field region, and a mean and standard deviation model of the DVH for the other OAR subregions.^34^, ^35^ When applying the RapidPlan model to a new patient, their geometric data (target and OAR contours, and field geometry) are used to predict a DVH curve for each OAR subregion, which are added together and weighted by the corresponding relative volume of each subregion. In this study, the OARs trained were the ipsilateral and contralateral breast, ipsilateral lung, and heart.

Within the RapidPlan workspace, a statistical summary describing the quality of the model is produced as an output of model training. For each OAR, the model's goodness‐of‐fit was evaluated using the coefficient of determination (*R* ^2^) and the average chi‐square (*χ* ^2^; related to Pearson's chi‐squared test) for the regression model parameters. *R* ^2^ and *χ* ^2^ values that are closer to one indicate a better model fit. Potential geometric and dosimetric outliers in the training dataset were identified using the regression and residual plots, and Cook's distance.^34^, ^35^ Regression plots show the first DVH principal component score as a function of the most important geometric parameter. Residual plots show the first principal component score of the actual DVH as a function of the first principal component score of the estimated DVH. Any training case deviating from the general model behavior was considered a potential outlier. Potentially influential cases were determined using Cook's distance, which measures the impact a single case has on the regression coefficients. Influential cases may not necessarily appear as an outlier in the regression and residual plots. A Cook's distance greater than 4 indicates an influential case that may be a geometric or dosimetric outlier. All cases identified as potential outliers were carefully reviewed and found to have anatomical differences with respect to the rest of the population in the training dataset. As a result, no patients were excluded from the training dataset to better capture the variation in patient anatomy.

The iterative process described by Hussein et al.^25^ was performed to refine the optimization objective parameters. Tables 2 and 3 summarize the final optimization objectives defined in the RapidPlan model. The final values are a result of iterative testing of the model performance on a randomly selected subset of 20 patients from the validation dataset. Values were tuned to obtain plans compliant with our institution's acceptance criteria and strategies concerning the trade‐offs between target coverage and OAR sparing. The MLC modulation *X* and *Y* smoothing parameters in the Photon Optimizer were set to *X* = 50 and *Y* = 40. Target coverage, uniformity, and conformity were improved by increasing the priority of the upper and lower target objectives and changing the normal tissue objective to our local optimized settings. For OARs, line‐type objectives with generated priorities are placed along the inferior border of the predicted DVH range. In some patients where the target was located in an inner breast quadrant, it was found that the heart and lung DVHs were overestimated by RapidPlan. Additional point objectives were added for these organs so that in the event of any cases where RapidPlan estimated a higher value for the line objective than the fixed point objectives, then the point objectives would take priority and achieve better sparing. In the following sections, the results refer to the final refined RapidPlan model.

**TABLE 2:** Post‐refinement RapidPlan model optimization objectives and priorities

**Structure**	**Type**	**Volume (%)**	**Dose (cGy)**	**Priority**
DEV	Lower	100	2700	160
PTV	Upper	0	2775	160
	Lower	98	2700	160
Ipsilateral breast	Line	Generated	Generated	Generated
Contralateral breast	Line	Generated	Generated	Generated
Ipsilateral lung	Line	Generated	Generated	Generated
	Upper	15%	270	120
	Upper	5%	810	120
Heart	Line	Generated	Generated	Generated
	Upper	5%	135	120

**Abbreviation**: DEV, dose evaluation volume, PTV, planning target volume “Generated” means the value was generated automatically by RapidPlan.

**TABLE 3:** Normal tissue objective (NTO) parameters used in the RapidPlan model

NTO parameter	Value
Priority	180
Distance from target border (cm)	0.20
Start dose (%)	95.0
End dose (%)	40.0
Fall‐off	0.09

### 2.3 Model validation

RapidPlan model performance was evaluated using 120 consecutively treated APBI patients that were not included in the training dataset. Herein, RapidPlan‐optimized plans will be denoted as KB plans. The clinical and KB plans were generated with the same prescription dose, beam energy and geometry, and optimization (Photon Optimizer, version 15.6.06) and dose calculation algorithms (Anisotropic Analytical Algorithm, version 15.6.06). All dose calculations were performed on a 2.5 mm grid with heterogeneity corrections turned on. To facilitate dosimetric comparison, all plans were normalized such that 98% of the DEV received 95% of the prescription dose.

### 2.4 Dosimetric evaluation

The dosimetry of KB plans was compared to clinical plans, and both the clinical^30^ and phase III trial^5^, ^6^, ^7^ planning constraints. The conformity index (CI) was used to score the conformity of the high dose region to the target. The CI is defined as the volume of tissue receiving at least 95% of the prescription dose divided by the PTV volume. Dose homogeneity within the target was assessed using the near‐maximum dose (D1cc) to the PTV. Differences between clinical and KB plans were evaluated using paired two‐tailed Wilcoxon signed‐rank tests (*α* = 0.05). The Benjamini–Yekutieli procedure was used to correct p‐values for multiple comparisons. All statistical tests were performed using SciPy 1.0 ^36^ in Python.

## 3 RESULTS

The characteristics of patients included in the training and validation datasets are provided in Table 4.

**TABLE 4:** Patient characteristics in the training and validation datasets

**Patient characteristic**	**Training dataset, count or median (range)**	**Validation dataset, count or median (range)**
Number of patients	64	120
Seroma volume (cm^3^)	8.6 (1.0–49.9)	7.2 (1.3–114.9)
PTV volume (cm^3^)	106.6 (21.4–288.1)	121.9 (59.2 419.8)
Ipsilateral breast volume (cm^3^)	1548.3 (497.6–3923.5)	1380.4 (507.6–4216.5)
Left‐sided laterality	40	69
Lower inner	8	10
Lower outer	19	16
Upper inner	7	22
Upper outer	6	21
Right‐sided laterality	24	51
Lower inner	3	5
Lower outer	5	15
Upper inner	10	14
Upper outer	6	17

**Abbreviation**: PTV,planning target volume.

**FIGURE 1:**
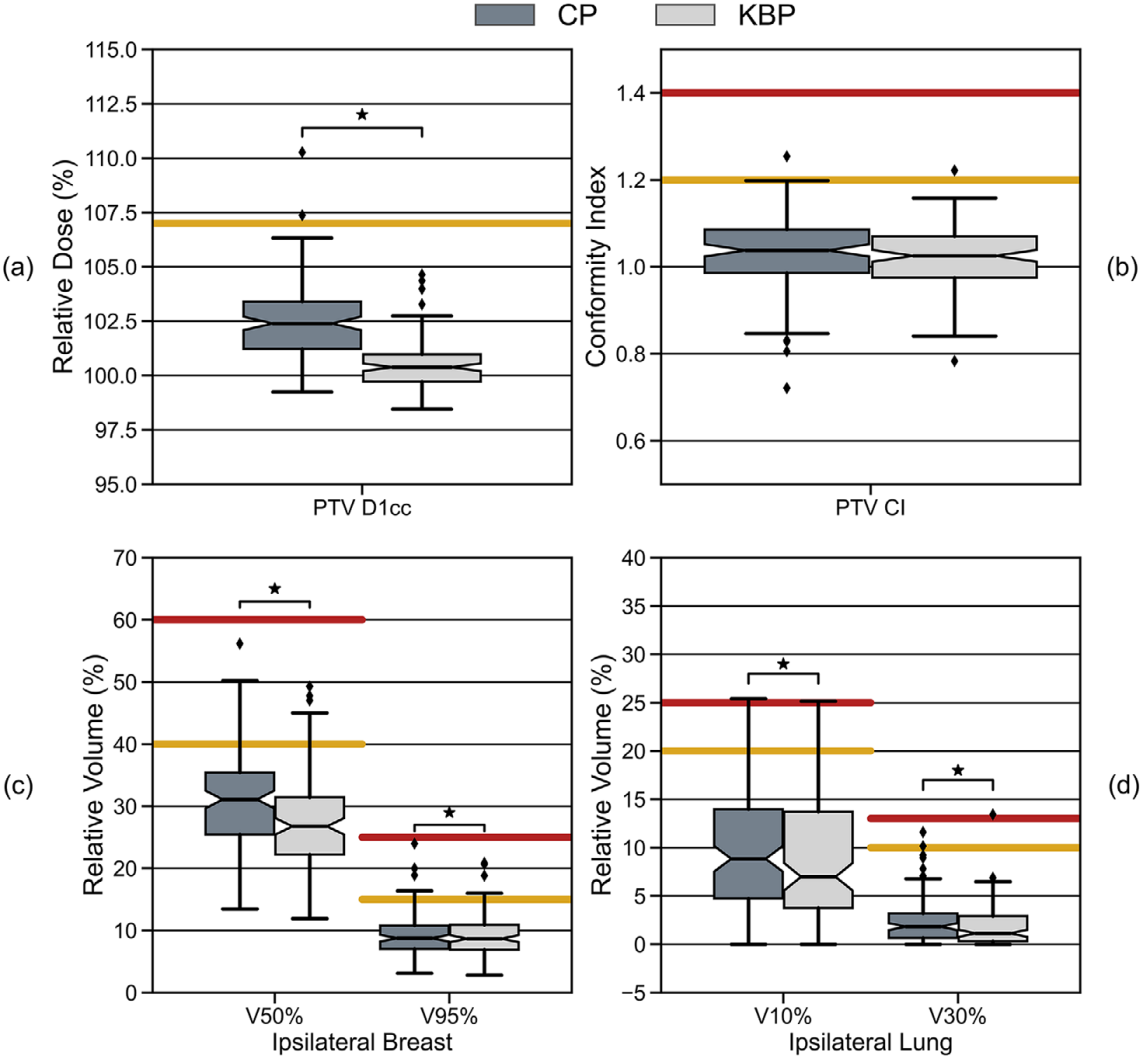
Comparison of the (a) planning target volume (PTV) D1cc; (b) PTV conformity index (CI); (c) ipsilateral breast V50% and V95%; and (d) ipsilateral lung V10% and V30% for clinical plans (CP: dark gray) and knowledge‐based plans (KBP: light gray). The yellow and red reference lines indicate the clinical planning constraints and the upper bounds of the minor variations respectively (see Table 1). Wilcoxon signed‐rank tests show that differences between CP and KBP are statistically significant for the PTV D1cc; ipsilateral breast V50% and V95%; and ipsilateral lung V10% and V30%

Figure 1a,b compares the PTV D1cc and CI, respectively, between clinical and KB plans in the validation dataset. A statistically significant improvement in PTV dose homogeneity was observed (median difference: 2.0%, range: −1.6%–7.6%, *p* < 0.05) for KB plans compared to clinical plans. All PTV D1cc values in the KB plans meet the planning constraint of D1cc < 107%. The PTV CI is comparable between clinical and KB plans (median difference: 0.0, range: −0.1–0.2, *p* = 0.05), and are within the planning constraints or minor variations.

The ipsilateral breast volume receiving 50% and 95% of the prescription dose for clinical and KB plans are plotted in Figure 1c. All ipsilateral breast V50% and V95% values are within the planning constraints or minor variations. The KB plans provided a statistically significant improvement in the ipsilateral breast V50% (median difference: 3.2%; range: −1.2%–17.4%; *p* < 0.05) and V95% (median difference: 0.1%; range: −1.3%–3.3%; *p* < 0.05) compared to the clinical plans.

The ipsilateral lung volume receiving 10% and 30% of the prescription dose for clinical and KB plans are plotted in Figure 1d. KB planning improved the lung V10% (median difference: 1.2%; range: −5.7%–5.9%; *p* < 0.05) and V30% (median difference: 0.4%; range: −2.3%–4.0%; *p* < 0.05) compared to conventional inverse planning for most patients.

The median (range) contralateral breast V3% in the clinical and KB plans were 0.0% (0.0%–1.6%) and 0.0% (0.0%–1.9%), respectively (median difference: 0.0%, range: −0.3%–0.4%, *p* = 1.00). For all right‐sided cases, the heart V5% was 0% in clinical and KB plans. For left‐sided lower inner quadrant cases, the median (range) heart V15% in the clinical and KB plans were 0.0% (0.0%–3.5%) and 0.0% (0.0%–3.8%), respectively (median difference: 0.0%, range: −0.3%–1.3%, *p* = 0.44). For all other quadrants in left‐sided cases, the median (range) heart V10% were 0.0% (0.0%–1.9%) and 0.0% (0.0%–1.1%) in the clinical and KB plans respectively (median difference: 0.0%, range: 0.0%–0.9%, *p* = 0.06). All clinical and KB plans met the planning constraints for the contralateral breast and heart, and differences were not statistically significant.

## 4 DISCUSSION

Variation in the technical details, efficacy, and cosmesis results of phase III APBI trials have left the community with questions regarding the optimal prescription dose and fractionation, treatment modality, patient selection, and volume to irradiate.^16^ Due to the confounding nature of these variables, the consequences of the selected dose, fractionation, and patient population will likely be clearer if dosimetry is well‐controlled. In this study, our institutional experience in APBI was used to train and validate a RapidPlan model. The RapidPlan model is able to produce IMRT plans in a single optimization without manual intervention that are comparable or better than the conventionally optimized clinical plans.

KB planning provided a statistically significant reduction in the PTV D1cc compared to conventional inverse planning. Previous studies have shown that the delivery of excessive radiation dose to regions within the breast is associated with acute and chronic toxicities and less optimal cosmetic results.^37^, ^38^ The maximum PTV D1cc values in KB and clinical plans were 104.6% and 110.3%, respectively. The improvement in dose homogeneity with KB planning is unlikely to be clinically significant but KB planning consistently met the planning requirement of D1cc < 107%.^30^

KB and conventional inverse planning achieved similar PTV CIs, with all plans fulfilling the planning constraints or minor variations.^30^ The conformity of the KB and clinical plans was comparable and has previously been reported^30^ to be superior to that accepted in recently published phase III APBI trials.^5^, ^6^ Early results from the ACCEL trial report favorable toxicity and cosmesis for this planning strategy.^39^

The difference in the ipsilateral breast V95% between KB and clinical plans was statistically significant, but small, with a median difference < 1%. Small differences in ipsilateral breast V95% between KB and clinical plans are expected because dose conformity for APBI has a stronger dependence on patient anatomy and the IMRT beam arrangement.^29^ In contrast, the ipsilateral breast V50% was significantly reduced in the KB plans, with a maximum difference of 17.4%. Several studies have investigated the relationship between ipsilateral breast dose and normal tissue toxicity or cosmesis after APBI.^13^, ^14^ Statistically significant associations have been demonstrated between the ipsilateral breast V50% and the risk of grade 2–4 subcutaneous fibrosis, fat necrosis, and fair/poor cosmetic outcome.^13^, ^14^ These relationships suggest that stricter dose constraints for the ipsilateral breast may be appropriate and can consistently be achieved by implementing the RapidPlan model.

Early‐stage breast cancer patients have excellent long‐term breast cancer‐specific survival and it is critical to minimize normal tissue doses to reduce the risk of long‐term toxicities. Heart and contralateral breast doses in KB plans were not significantly different compared to clinical plans, and the ipsilateral lung dose was lower in KB plans for most patients. In general, APBI provides very low normal tissue doses and dose–volume parameters for the ipsilateral lung, heart, and contralateral breast are substantially lower than in WBI^29^ or QUANTEC recommendations.^40^, ^41^

The large‐scale retrospective application of our RapidPlan model for IMRT‐based APBI has demonstrated the model's validity for clinical implementation. This RapidPlan model has several potential applications. RapidPlan models can be shared with other institutions, providing an opportunity to transfer planning expertise from our more experienced center to less experienced centers looking to adopt APBI.^26^, ^29^, ^30^, ^42^ This RapidPlan model could be supplied to participating centers for future multi‐institutional clinical trials to standardize the planning technique and dosimetric criteria^29^, ^30^ so that they are better positioned to answer remaining questions regarding optimal prescription dose, fractionation, and patient selection. Alternatively, the RapidPlan model could be used as a tool for planning quality assurance in clinical trial treatment plan audit/credentialing and to benchmark different techniques.^42^, ^43^, ^44^ Tol et al.^42^ proposed a workflow utilizing RapidPlan to quickly evaluate whether a treatment plan submitted to a clinical trial provides sufficient OAR sparing by comparing the submitted plan OAR DVHs to those predicted by RapidPlan. Overall, clinical implementation of this RapidPlan model for treatment planning or as a quality assurance tool provides the opportunity to standardize plan quality and could benefit any future phase III APBI trials looking to address the shortcomings of previous trials.

## 5 CONCLUSION

A RapidPlan model was successfully trained and validated for left‐ and right‐sided APBI planning. Model validation results show an improvement in plan quality and consistency for KB plans compared to conventionally optimized clinical plans. These results suggest that this model can be reliably applied in clinical practice and may be used to transfer planning expertise to ensure consistency in APBI plan quality.

## CONFLICT OF INTEREST

The authors declare no conflict of interest.

## FUNDING INFORMATION

None.

## AUTHOR CONTRIBUTIONS

Amy Frederick and Sarah Quirk conceived the project. Amy Frederick developed and validated the RapidPlan model, and collected and analyzed the data. Michael Roumeliotis, Petra Grendarova, and Sarah Quirk provided clinical expertise and supervision of the project. Amy Frederick drafted the manuscript and all co‐authors revised and approved the final manuscript.

## Data Availability

The RapidPlan model and relevant data are available from the authors upon reasonable request. S. Quirk can be contacted to request data.
